# 900 MHz Radiofrequency Field Induces Mitochondrial Unfolded Protein Response in Mouse Bone Marrow Stem Cells

**DOI:** 10.3389/fpubh.2021.724239

**Published:** 2021-08-26

**Authors:** Wen Xie, Rui Xu, Caiyun Fan, Chunyu Yang, Haiyan Chen, Yi Cao

**Affiliations:** ^1^Department of Toxicology, School of Public Health, Medical College of Soochow University, Suzhou, China; ^2^Jiangsu Key Laboratory of Preventive and Translational Medicine for Geriatric Diseases, Suzhou, China

**Keywords:** mitochondria, radiofrequency fields, unfolded protein response, reactive oxygen species, microwave, heat shock protein

## Abstract

**Objective:** To examine whether exposure of mouse bone marrow stromal cells (BMSC) to 900 MHz radiofrequency fields used in mobile communication devices can induce mitochondrial unfolded protein response (UPR^mt^).

**Methods:** BMSCs were exposed to continuous wave 900 MHz radiofrequency fields (RF) at 120 μW/cm^2^ power intensity for 4 h/d for 5 consecutive days. Cells in sham group (SH) were cultured in RF exposure system, but without RF radiation. The positive control cells were irradiated with 6 Gy X-ray at a dose rate of 1.103 Gy/min (XR). To inhibit the upstream molecular JNK2 of UPR^mt^, cells in siRNA + RF, and siRNA + XR group were also pretreated with 100 nM siRNA-JNK2 for 48 h before RF/XR exposure. Thirty minutes, 4 h, and 24 h post-RF/XR exposure, cells were collected, the level of ROS was measured with flow cytometry, the expression levels of UPR^mt^-related proteins were detected using western blot analysis.

**Results:** Compared with Sham group, the level of ROS in RF and XR group was significantly increased 30 min and 4 h post-RF/XR exposure (*P* < 0.05), however, the RF/XR-induced increase of ROS level reversed 24 h post-RF/XR exposure. Compared with Sham group, the expression levels of HSP10/HSP60/ClpP proteins in cells of RF and XR group increased significantly 30 min and 4 h post-RF/XR exposure (*P* < 0.05), however, the RF/XR-induced increase of HSP10/HSP60/ClpP protein levels reversed 24 h post-RF exposure. After interfering with siRNA-JNK2, the RF/XR exposures could not induce the increase of HSP10/HSP60/ClpP protein levels any more.

**Conclusions:** The exposure of 900 MHz RF at 120 μW/cm^2^ power flux density could increase ROS level and activate a transient UPR^mt^ in BMSC cells. Mitochondrial homeostasis in term of protein folding ability is restored 24 h post-RF exposure. Exposure to RF in our experimental condition did not cause permanent and severe mitochondrial dysfunctions. However, the detailed underlying molecular mechanism of RF-induced UPR^mt^ remains to be further studied.

## Introduction

Non-ionizing radiofrequency fields (RF) are ubiquitous in the environment. They are used in military, radio, and television broadcasting, wireless communications systems, industry, and medicine. The number of people exposed to RF increased exponentially with the introduction of wireless communication devices which transmit voice, data and images. The scientific information on the biological and health effects of exposure to RF is more extensive now than ever before (1). Nonetheless, the unfolded protein response (UPR) in mitochondria in cells exposed to RF received little attention of researchers. Mitochondria is the organelle with the closed bilayer membranes structure, which play a key role in cellular biosynthetic, intracellular oxidative phosphorylation and the regulation of calcium levels. Mitochondria contain specific heat shock proteins (HSP) and protease, which help to fold, unfold, or degrade other proteins for the protein equilibrium inside. When a large number of unfolded or misfolded proteins are accumulated in cells due to external stimulus, the reverse signaling pathway from mitochondria to nucleus will be activated to increase the expression of nuclear genes encoding mitochondrial proteins (2). The newly-synthesized mitochondrial proteins include chaperones HSP10 and HSP60 which facilitate the import and correct folding of unfolded proteins, and proteases ClpP and ClpX which help to degrade the unfolded and misfolded proteins. This process is called mitochondrial unfolded protein response (UPR^mt^) (3–7).

Mitochondria are the main source and one of the targets of RF-induced reactive oxygen species (ROS) (8). Electromagnetic field (EMF) directly targets the electron transport chain, which leads to mitochondrial dysfunction and overproduction of ROS. EMF can cause a disturbance of mitochondrial proton motive force, which then disrupts the balance between ROS production and ROS clearance. The severe and long-lasting oxidative stress may play a key role in mitochondrial damage that leads to some human health problems (9). However, mild ROS increase could lead to cellular defense mechanisms, including unfolded protein response (10). Studies showed that during mild mitochondrial dysfunction, UPR^mt^ activation promotes development and prolonging longevity, suggesting that UPR^mt^ activation may be a useful therapeutic approach for some mitochondria-related diseases. However, prolonged or dysregulation of UPR^mt^ activation can exacerbate mitochondrial dysfunction caused by external stimuli (11–13).

Currently, there are few reports on the effect of RF-induced ROS on UPR^mt^. In view of the paucity of RF investigations on UPR in mitochondria, we have conducted the present study to investigate whether 900 MHz RF can induce UPR^mt^ in BMSCs. Mouse bone marrow stem cells (BMSCs) were exposed to 900 MHz RF for 4 h/d for 5 d to examine if RF exposure can induce UPR in mitochondria. Sham-exposed (SH) control cells as well as those exposed to an acute dose of ionizing/X-rays radiation (XR) as positive control cells were included in the experiment. The expression levels of HSP10/HSP60/ClpP proteins involved in UPR^mt^ were examined. To verify the induction of UPR in BMSCs, siRNA-JNK2 was used to inhibit the known signal molecular JNK2 of UPR^mt^ in the study.

## Materials and Methods

### Bone Marrow Stromal Cells

The collection and culture of BMSCs were described in detail in our earlier paper (14). Single cell suspensions were prepared in complete IMDM medium (Iscove's modified Dulbecco's medium, Hyclone, Suzhou, China) containing 10% fetal bovine serum (FBS, Gibco, Shanghai, China), 100 units/ml penicillin and 100 μg/ml streptomycin (Bio Basic, Hangzhou, China). From each mouse, aliquots of 4 × 10^5^ cells in 10 ml medium were placed in 100 mm petri dishes cultured in an incubator (Heal Force Bio-Meditech, Hong Kong, China) maintaining 37 ± 0.5°C with humidified atmosphere of 95% air and 5% carbon dioxide (CO_2_). Cells in 3–6 passages from a single mouse were used for different exposures described below.

### Radiofrequency Fields/Sham Exposed Exposure

The exposure system was built in-house and consists of a signal generator (SN2130J6030, PMM, Cisano sul Neva, Italy), a power amplifier (SN1020, HD Communication, Ronkonkoma, NY), and a Gigahertz Transverse Electro-Magnetic (GTEM) chamber. The RF signal was generated, amplified and fed through an antenna (Southeast University, Nanjing, Jiangsu, China) and detected by a field strength meter (PMM, Cisano sul Neva, Italy). The specific operation principle and exposure protocols has been discussed in detail by the previous studies of our lab (14–16). The same GTEM without RF transmission, was used for SH-exposure of cells. During the RF/SH exposure, the culture medium was changed once and the external environment was maintained 37 ± 0.5°C with 87% relative humidity (without CO_2_). The peak and average SARs could be computed by either frequency or time domain method (17), and the estimated values were extremely low: they were 4.1 × 10^−4^ and 2.5 × 10^−4^ W/kg, respectively.

### X-Ray Radiation

Irradiations were performed with an X-ray apparatus (Rad Source Technologies Inc., USA) operating at a dose rate of 1.103 Gy/min.

### Group Design

Several 100 mm petri dishes, each containing ~4 × 10^5^ cells/ml (total 10 ml medium), were used for the following exposure conditions: (a) kept in GTEM without RF (Sham, SH); (b) 900 MHz RF, 120 μW/cm^2^ power intensity for 4 h/d for 5 d (RF); (c) acute 6 Gy X-ray radiation (XR); (d) kept in GTEM without RF for 4 h/d for 5 d after siRNA transfection (si + SH); (e) 900 MHz RF for 4 h/d for 5 d after siRNA transfection (si + RF); (f) acute 6 Gy X-ray radiation after siRNA transfection (si + XR). Cells in each group were collected at 30 min, 4 h, and 24 h post treatment for subsequent experiments. The entire investigation was repeated 3 times.

### Measurement of Reactive Oxygen Species

ROS level was measured with reactive oxygen detection kit (Beyotime, Shanghai, China). After different treatments, the cells were gently washed twice with neutral phosphate buffer after digestion and centrifugation. Serum-free IMDM medium (Hyclone, USA) was used to prepare DCFH-DA solution with a final concentration of 10 μM. One mL of DCFH-DA solution was added into the centrifuge tubes of each group and thoroughly mixed. Centrifuge tube was incubated at 37°C for 20 min, mixed upside down every 3 min to promote maximum contact between the cells and the probe. At the end of incubation, supernatant was discarded, and the cells were washed with serum-free IMDM medium for 3 times. Then, the intracellular fluorescence intensity was measured at excitation wavelength of 488 nm and the emission wavelength of 525 nm.

### siRNA Transfection

Transfection with siRNA was used to inhibit JNK2 expression, in a separate series of experiments, prior to sham, RF and X-rays exposure, cells grown to 60–70% confluence were transfected with 100 nM siRNA (ribo FECT^−^ CP, Guangzhou, China) for 48 h, the target sequence used for JNK siRNA were GGCATCAAGCATCTGCATT. Subsequently, siRNA was washed out three times followed by the different exposure conditions. Transfection was performed for 48 h, and then the cells were collected. Quantitative real-time PCR was used to verify the transfection efficiency.

### Western-Blot Analysis

In this study, HSP60/HSP10/ClpP protein, known UPR^mt^ markers, were chosen as the indicators to verify whether UPR^mt^ occurred (18–20). The expression levels of the heat shock protein HSP60/HSP10 and mitochondrial protease ClpP were detected with Western-Blot Analysis. Protein extracts were prepared by lysing the cells in lysis buffer containing 50 mM Tris (pH 7.4), 150 mM sodium chloride, 1%Triton X-100, 1% sodium deoxycholate, 0.1% sodium dodecyl sulfate, and 1 mM phenyl-methyl-sulfonyl fluoride (all obtained from Beyotime, Shanghai, China). The cell lysates were centrifuged at 14,000 × g for 5 min at 4°C and the supernatant containing solubilized proteins was collected. The protein concentration in all samples was determined using the BCA protein assay kit (Beyotime, Shanghai, China). From each sample, equal amount of protein (40 μg per lane) was loaded, separated by 10% sodium dodecyl sulfate polyacrylamide gel (SDS–PAGE) and then transferred to polyvinylidene difluoride (PVDF) membranes (Millipore Corporation, Billerica, MA, USA). The membranes were blocked for 2 h in 5% fat-free dry milk (Yili Industrial, Inner Mongolia, China) containing Tris Buffered Saline with Tween (TBST). The membranes were then incubated with primary antibodies (rabbit monoclonal anti-HSP10 antibody, rabbit monoclonal anti-HSP60 antibody, rabbit monoclonal anti-ClpP antibody, and rabbit monoclonal anti-GADPH, Abcam, Cambridge, USA) overnight at 4°C. They were washed three times in TBST and incubated further with horseradish peroxidase-conjugated antibodies (Beyotime, Shanghai, China) for 1.5 h at room temperature. This was followed by washing the membranes three times with TBST. The immunoreactive proteins on the membranes were detected using enhanced chemiluminescence reagents (Millipore Corporation) and G-BOX Chemi XRQ (Syngene, UK). The blots were quantified and normalized with the level of GADPH to correct the differences in loading of the proteins in different treatment cells.

### Statistical Analyses

The results from three independent experiments were pooled and analyzed using GraphPad Prism 8.0 (GraphPad Software, San Diego, CA, USA). The results were subjected to One-way analysis of variance (ANOVA) to test differences between groups. A *P* < 0.05 was considered as significance difference between groups.

## Results

### Reactive Oxygen Species

**Figure 1:**
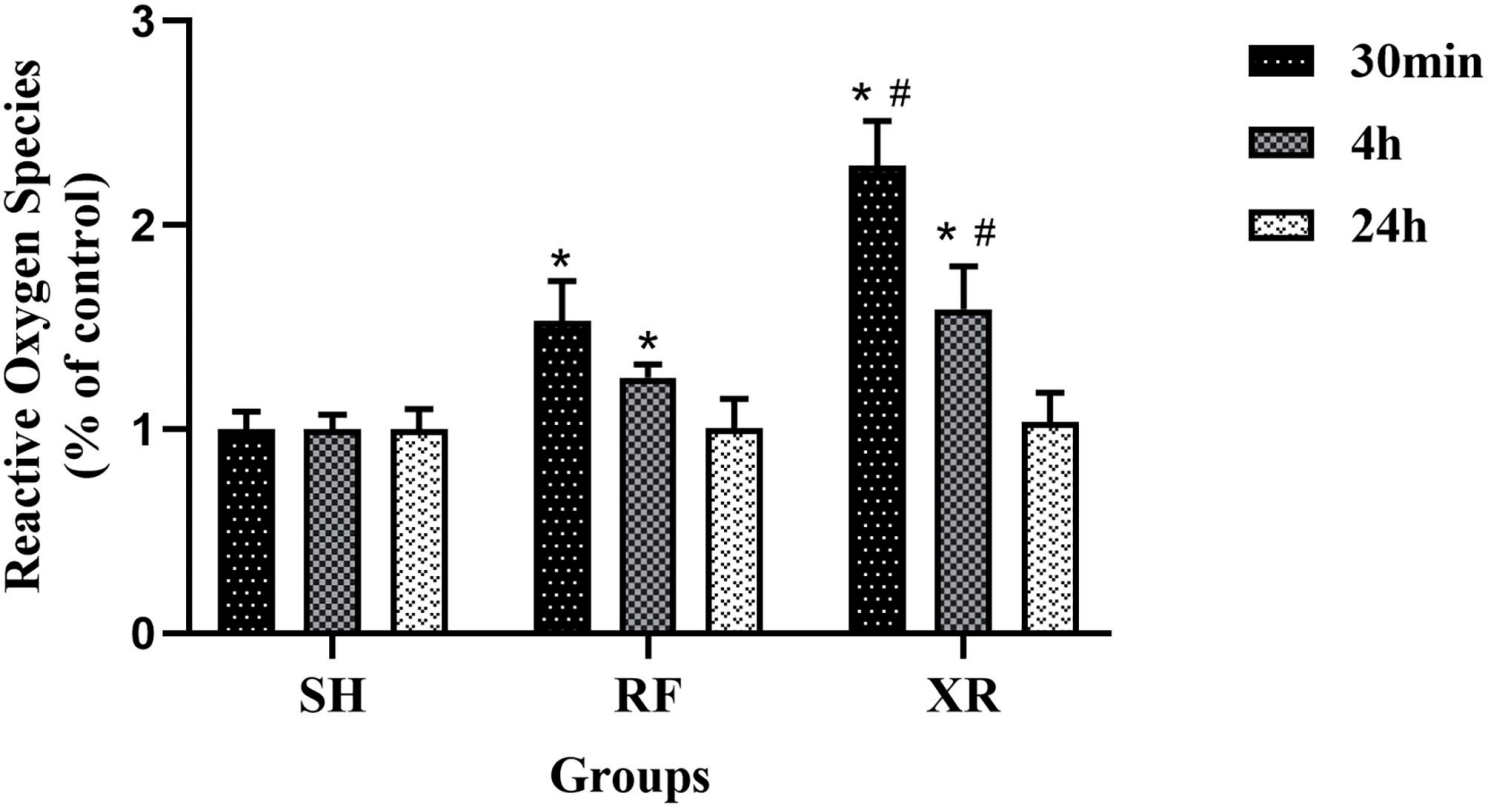
ROS levels in BMSCs at 30 min, 4 h, and 24 h post exposure. SH, Sham; RF, RF-exposed; XR, X-rays irradiated. RF and XR vs. SH: ^*^P < 0.05; XR vs. RF: ^#^*P* < 0.05.

The expression levels of ROS in different groups were shown in Figure 1. The level of ROS in the RF group and XR group was significantly increased at 30 min and 4 h post-exposure (*P* < 0.05). The ROS level decreased to nearly those in SH cells 24 h post-RF and XR exposure. Compared with the cells exposed to RF, those exposed to XR showed significant increase in ROS at 30 min, 4 h (*P* < 0.05). These results indicated that both RF and X-ray could induce the production of ROS in BMSCs for a certain period of time. The increase of ROS induced by X-ray was much higher than that induced by RF.

### siRNA Transfection Efficiency

**Figure 2:**
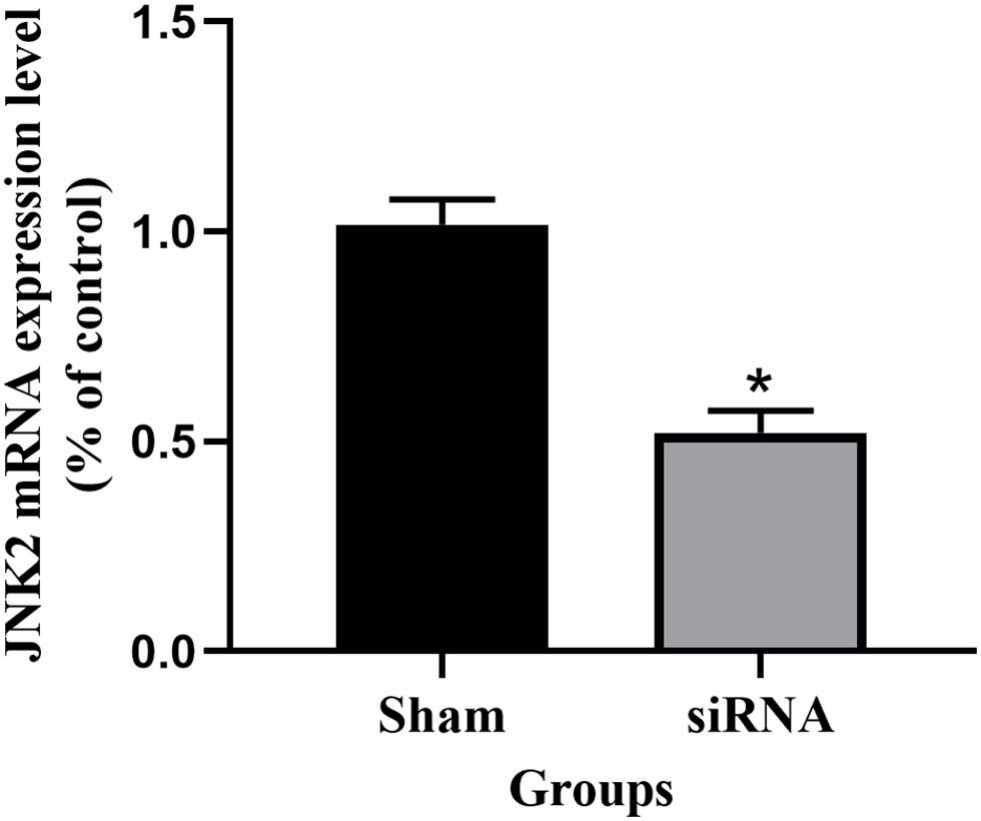
The expression level of JNK2 mRNA after siRNA transfection for 48 h. siRNA vs. Sham: ^*^P < 0.05.

After siRNA transfection for 48 h, the expression level of JNK2 mRNA was shown in Figure 2. The expression level of JNK2 mRNA decreased significantly compared to sham group (*P* < 0.05). The knockdown efficiency of siRNA transfection is 50%.

### HSP10, HSP60, and ClpP Proteins (Western Blot Analysis)

**Figure 3:**
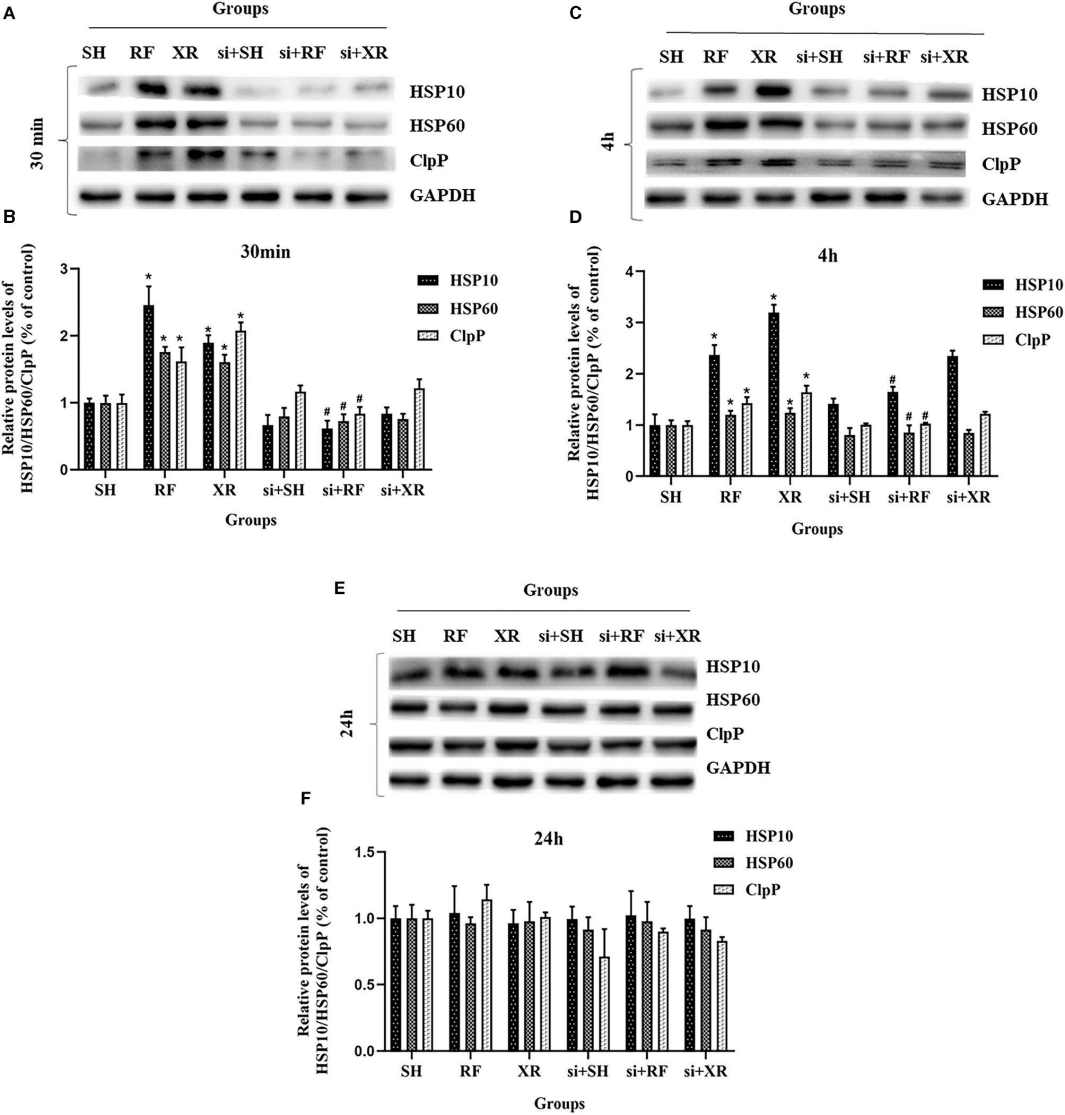
Protein levels of HSP10, HSP60, ClpP in BMSCs after exposure. **(A)** Western blot analysis of HSP10, HSP60, ClpP in BMSCs at 30 min post exposure. **(B)** Relative expression on level of HSP10, HSP60, ClpP protein at 30 min post exposure. **(C)** Western blot analysis of HSP10, HSP60, ClpP in BMSCs at 4 h post exposure. **(D)** Relative expression on level of HSP10, HSP60, ClpP protein at 4 h post exposure. **(E)** Western blot analysis of HSP10, HSP60, ClpP in BMSCs at 24 h post exposure. **(F)** Relative expression on level of HSP10, HSP60, ClpP protein at 24 h post exposure. SH, Sham; RF, RF-exposed; XR, X-rays irradiated; si + SH, siRNA + Sham; si + RF, siRNA + RF-exposed; si + XR, siRNA + X-rays irradiated. RF and XR vs. SH: ^*^P < 0.05; si + RF vs. RF: ^#^*P* < 0.05.

The expression levels of HSP10, HSP60, ClpP proteins in different groups at 30min, 4 h, and 24 h were shown in Figure 3. Compared to the cells in SH group, the expression levels of HSP10, HSP60, ClpP proteins in the RF group increased significantly at 30 min and 4 h (*P* < 0.05), then decreased gradually and returned to nearly those in SH cells at 24 h. These results indicate that low-dose Radio-frequency could induce UPR^mt^ in BMSCs. After the interference of the upstream molecular JNK2 of UPR^mt^ with siRNA, the expression levels of HSP10, HSP60, ClpP proteins in si + RF group were significantly decreased compared to the RF group (*P* < 0.05), indicated that RF activates UPR^mt^ through the JNK2 signaling pathway.

## Discussion

Mitochondria are organelles that play important functions in cells and participate in a variety of physiological functions and biochemical reactions, including ATP production, iron-sulfur cluster biosynthesis, nucleotide and amino acid metabolism, and cell apoptosis (21–24). In order to maintain normal cellular physiological activities and functions, mitochondria have a complete set of molecular chaperone systems and quality control proteases, which promote the correct folding of proteins and degradation of misfolded/unfolded proteins (25, 26). Upon environmental stress, the accumulation of unfolded protein in the mitochondria could reach a certain threshold or severely damaged mitochondria appear in the cell, hence UPR^mt^ can enhance the overall function of cellular mitochondria by upregulating protective molecular chaperones and proteases that promote protein folding or clearance of defective proteins within stressed mitochondria, restore and maintain mitochondria steady state, and ultimately prevent and/or reduce the damage of environmental factors to the cells.

Non-ionizing radiofrequency fields (RF, 300 MHz to 300 GHz) are ubiquitous in environment (27). 900 MHz RF is one of the frequencies commonly used in mobile communication. High doses or long duration of radiofrequency radiation will exert harmful health effects on living organisms. The power density of 120 μW/cm^2^ used in this study is below the exposure limit stipulated by the International Commission on Non-Ionizing Radiation Protection (ICNIRP), which is generally considered as low dose radiation. The biological effects and health impacts of long-term exposure to low-dose RF are a matter of widespread concern, and also a scientific issue that needs to be studied (28). In this study, we found that exposure to RF in our experimental condition did not cause permanent and severe mitochondrial dysfunctions. Our findings may shed light on dark areas of health effects of human exposure to radiofrequency radiation.

To the best of our knowledge, there have been very few peer-reviewed scientific publications in which ROS and mitochondrial UPR were examined in cells exposed to RF. Studies have shown that RF radiation can induce oxidative damage of mtDNA in cerebral cortical neurons of SD rats (29). It is reported that mitochondrial UPR could inhibit oxidative phosphorylation and ROS production (30). Increased protein level of chaperones HSP10, HSP60, and protease ClpP are considered to be the hallmark of UPR^mt^ activation (2, 3). As a mitochondrial chaperone protein encoded by the nuclear genome, HSP60 mainly promotes the folding of relatively small soluble monomeric proteins (31, 32), and plays an important role in protein transport and folding in the mitochondria. HSP10 is widely present in a variety of mammalian tissues and can bind to unfolded proteins, effectively reducing energy barriers that occur during protein folding (33, 34). Mitochondrial ClpP is a serine protease located in the mitochondrial matrix. ClpP forms a proteolytic complex with AAA+ partner ClpX called ATP-dependent unfolding enzyme (ClpXP), which degrades misfolded or denatured proteins to participate in the quality control of mitochondrial proteins and maintain normal metabolic function (35). In this study, the expression levels of HSP10, HSP60, and ClpP in BMSCs were all upregulated within a certain period of time upon radio-frequency and X-rays exposure, indicating that both RF and X-rays could induce UPR^mt^ in BMSCs, however, the level of unfolded protein response induced by ionizing X-rays radiation was much greater than non-ionizing radio frequency. This phenomenon possibly be explained by the more severe mitochondria damage and more misfolded protein aggregation caused by X-rays radiation.

Many literatures have reported that ionizing radiation and non-ionizing radiation could activate the JNK signaling pathway (36–39). The activation of JNK2 is closely related to the induction of UPR. Activation of JNK2 could trigger c-Jun binding to AP-1 elements to up-regulate CHOP and C/EBPβ transcription. Dimer of CHOP and C/EBPβ transcription factors binds to specific UPR^mt^ promoter element and activates the target genes (40, 41). In this study, after interfering with siRNA-JNK2, the RF/XR exposures could not induce the increase of HSP10/HSP60/ClpP protein level, suggesting that JNK2 is involved in 900 MHz RF-induced UPR^mt^. Our results are in agreement with reported investigations (36–41).

To further investigate an association between RF exposure and activation of UPR^mt^, the level of ROS after RF and X-rays exposure were measured. The rationale for the study is that RF-induced ROS possibly causes mitochondrial damage, and hence the accumulation of misfolded/unfolded proteins which in turn activate UPR^mt^ in BMSCs (2). One of the main sources of cellular ROS is the mitochondrial respiratory chain, when affected by external stimuli, ETC produces excessive ROS, disturbing the mitochondrial environment homeostasis (42, 43). When moderate ROS exists, it will play a beneficial physiological role, but when excessive ROS content increases, it will cause severe cell stress damage and even lead to cell death (44). For decades, people have been focused on the RF effect on the health of human body, many studies have tried to assess whether or not the RF can affect the production of ROS in cells. However, due to the differences in cell types, RF parameters and exposure time, etc., the effects of RF exposure on ROS levels are inconsistent (45). Some studies have shown that radio-frequency radiation could induce the increase of intracellular ROS content (46, 47). In this study, the increased ROS level are in line with the increased level of UPR^mt^-associated mitochondrial chaperone and protease. To date, very little is known about the mechanism underlying RF-induced UPR^mt^. Some investigations found that ROS increase is closely related to the induction of UPR^mt^, and UPR^mt^ is in turn an indispensable and complex response that allows cells to buffer ROS (48, 49), suggesting that RF exposure could induce the occurrence of UPR^mt^ via ROS production (50). The results of this research is consistent with the above reports.

Mitochondrial homeostasis determined the fate of cells. The functioning of the mitochondria are in turn tightly aligned to energy transduction and to the control of calcium and redox stress homeostasis (51). The observations obtained in our current study in BMSCs indicated that non-ionizing 900 MHz RF exposure at 120 μW/cm^2^ power density was capable of inducing UPR^mt^ in response to mitochondria stress, increasing the expression of HSP10/HSP60/ClpP proteins, restoring mitochondrial homeostasis. Although RF exposure has already been demonstrated to activate UPR^mt^ in this study, the detailed underlying mechanism remains to be further studied.

## Data Availability Statement

The original contributions presented in the study are included in the article/supplementary material, further inquiries can be directed to the corresponding author/s.

## Author Contributions

WX and RX conceived the manuscript, did the experiments, and wrote the draft of the manuscript. CF, CY, and HC reviewed the manuscript. YC designed the experiment and critically revised the manuscript. All authors listed have made a substantial, direct and intellectual contribution to the work, and approved it for publication.

## Conflict of Interest

The authors declare that the research was conducted in the absence of any commercial or financial relationships that could be construed as a potential conflict of interest.

## Publisher's Note

All claims expressed in this article are solely those of the authors and do not necessarily represent those of their affiliated organizations, or those of the publisher, the editors and the reviewers. Any product that may be evaluated in this article, or claim that may be made by its manufacturer, is not guaranteed or endorsed by the publisher.
